# Compatibility of quantitative X-ray spectroscopy with continuous distribution models of water at ambient conditions

**DOI:** 10.1073/pnas.1815701116

**Published:** 2019-02-19

**Authors:** Johannes Niskanen, Mattis Fondell, Christoph J. Sahle, Sebastian Eckert, Raphael M. Jay, Keith Gilmore, Annette Pietzsch, Marcus Dantz, Xingye Lu, Daniel E. McNally, Thorsten Schmitt, Vinicius Vaz da Cruz, Victor Kimberg, Faris Gel’mukhanov, Alexander Föhlisch

**Affiliations:** ^a^Institute for Methods and Instrumentation for Synchrotron Radiation Research, Helmholtz Zentrum Berlin für Materialien und Energie, D-12489 Berlin, Germany;; ^b^Department of Physics and Astronomy, University of Turku, FI-20014 Turun Yliopisto, Finland;; ^c^European Synchrotron Radiation Facility 71, F-38043 Grenoble Cedex 9, France;; ^d^Institut für Physik und Astronomie, Universität Potsdam, D-14476 Potsdam-Golm, Germany;; ^e^Swiss Light Source, Photon Science Division, Paul Scherrer Institut, 5232 Villigen PSI, Switzerland;; ^f^Theoretical Chemistry and Biology, Royal Institute of Technology, SE-10691 Stockholm, Sweden;; ^g^Institute of Nanotechnology, Spectroscopy and Quantum Chemistry, Siberian Federal University, 660041 Krasnoyarsk, Russia

**Keywords:** structure of water, X-ray spectroscopy, continuous distribution model

## Abstract

The phase diagram of water harbors controversial views on underlying structural properties of its constituting molecular moieties, its fluctuating hydrogen-bonding network, as well as pair-correlation functions. In this work, long energy-range detection of the X-ray absorption allows us to unambiguously calibrate the spectra for water gas, liquid, and ice by the experimental atomic ionization cross-section. In liquid water, we extract the mean value of 1.74 ± 2.1% donated and accepted hydrogen bonds per molecule, pointing to a continuous-distribution model. In addition, resonant inelastic X-ray scattering with unprecedented energy resolution also supports continuous distribution of molecular neighborhoods within liquid water, as do X-ray emission spectra once the femtosecond scattering duration and proton dynamics in resonant X-ray–matter interaction are taken into account. Thus, X-ray spectra of liquid water in ambient conditions can be understood without a two-structure model, whereas the occurrence of nanoscale-length correlations within the continuous distribution remains open.

## Significance

Water is the matrix of life and behaves anomalously in many of its properties. Since Wilhelm Conrad Röntgen, two distinct separate phases have been argued to coexist in ambient water, competing with the alternative view of the single-phase liquid, footing on X-ray scattering experiment and theory. We conducted a quantitative and high-resolution X-ray spectroscopic multimethod investigation and analysis (X-ray absorption, X-ray emission, and resonant inelastic X-ray scattering). We find that all known X-ray spectroscopic observables can be fully and consistently described with continuous-distribution models of near-tetrahedral liquid water at ambient conditions with 1.74 ± 2.1% donated and accepted H-bonds per molecule.

Since the electronic structure of water molecules can support both twofold and fourfold coordination in their molecular interaction, both a view of continuous distribution of molecular moities (homogeneous view) (1–7) and a view of oscillations between separate distinct phases (heterogeneous view) (8–12) of liquid water can be envisaged. The heterogeneous view foots strongly on the consideration that in the supercooled regime, statistical response functions diverge at 228 K, introducing a liquid–liquid critical point that would terminate the transition line between high- and low-density liquid phases (13).

The possible heterogeneous picture with fluctuations between two classes applied in the supercooled regime has also been suggested to exist far up in temperatures (320 K) of the ambient regime (14). This suggestion contradicts the physical view that above a critical point, the system is homogeneous and free from the need for multiphase classification (5, 15). However, even such a homogeneous or continuous-distribution model does not exclude statistical variation: Ambient and supercooled water have been found to naturally undergo density fluctuations in single-phase simulations (15). The two-phase model of liquid water has been repeatedly promoted by the interpretation of X-ray spectroscopic findings (ref. 16 and references therein), but the spectra have also been interpreted on the basis of homogeneous water models (17–19). The mixture hypothesis proposed by W. C. Röntgen (20) was refuted in 1970 by H. Frank (21) after a parallel review of contemporary X-ray scattering and vibrational spectroscopy data.

## X-Ray Absorption Spectra

**Fig. 1.**
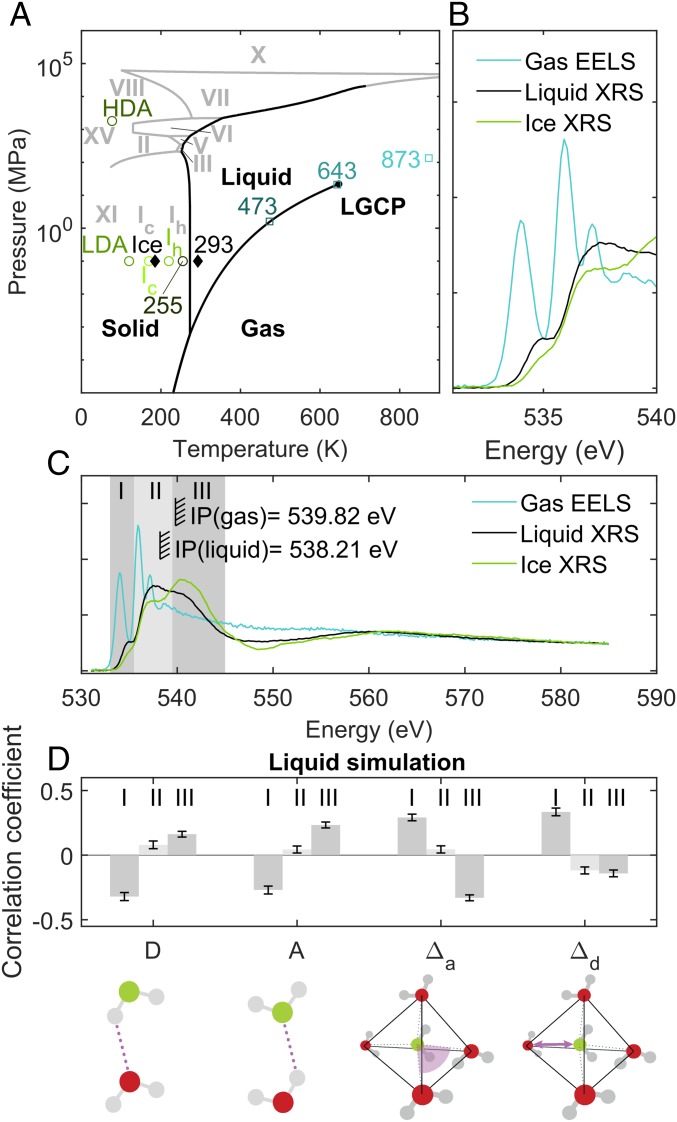
(*A*) Schematic phase diagram of water in relation to oxygen 1s XAS obtained from saturation-free hard X-ray Raman spectroscopy. (*B* and *C*) We replace the f-sum rule normalization with f-density (df(E)/dE) normalization at mean ionization cross-section between 580 and 585 eV for gas, liquid, and ice. Region I: LUMO 4$a_{1}$ preedge feature. Region II: Overlapping LUMO+1 2$b_{2}$ and continuum features. Region III: Continuum region, with shape resonance (${\text{hv}}_{in}∼$542 eV) from second-shell O–O continuum scattering resonance. (*D*) Line-intensity–structural-parameter correlation coefficients based on first-principles liquid simulation (30) for regions I–III (lesser correlations in *SI Appendix*, Fig. S4): donated (D) and accepted (A) hydrogen bonds, sum angular deviation from tetrahedrality ($\Delta_{a}$), and furthest-nearest difference ($\Delta_{d}$) for the closest four neighboring O sites.

Soft X-ray oxygen 1s X-ray absorption spectroscopy (XAS), electron energy loss spectroscopy (EELS), and equivalent information from hard X-ray Raman scattering (XRS) for the oxygen 1s excitations have been used to characterize the various phases of water (11, 22–27). In these studies, integral or area normalization within the measured spectral range between 530 and 550 eV has typically been used, with the aim to fulfill the theoretical concept of the f-sum rule (28) present for an ideal—complete—spectral range with clearly discernible bound and continuum states. Combining simulations with area normalization, a significant signature of broken hydrogen bonds in liquid water has been postulated (11) based on the observation of increasing intensity in the 4$a_{1}$ lowest unoccupied molecular orbital (LUMO) line (I in Fig. 1*C*) along transitions from ice to liquid water and finally the gas phase. However, in the f-sum rule normalization, the decrease of intensity in the preedge region is exactly counterbalanced by an intensity gain in other regions within the normalization range, because the integrated spectral intensity is forced to a fixed value. Therefore, using area normalization within the range of 530–550 eV to fulfill the f-sum rule induces a trade-off in spectral intensity within the experimentally accessed energy range. Moreover, this trade-off is limited to the normalization range, which is unjustified, as such spectra do not meet each other at the end of the integration interval (*SI Appendix*, Fig. S1).

In Fig. 1 *A*–*C*, we present the phase diagram of water in relation to oxygen 1s XAS spectra obtained from saturation-free hard X-ray Raman spectroscopy for ice and liquid and EELS for gas from ref. 22 (raw data after subtraction of constant background), where we follow the idea presented in refs. 27 and 29 and replace the f-sum rule normalization with f-density (df(E)/dE) normalization at the high-energy end (see also *SI Appendix*, Fig. S2). Fig. 1 *B* and *C* represent scans of gas, liquid, and ice. The use of f-density normalization is based on the reasoning that at the sudden limit (a fast photoelectron), the photoionization cross-section is an atomic property, independent of sample composition and varying bonding situations. Thus, f-density links XAS state populations of different materials and molecules via fundamental core-continuum transition properties in the most reliable way when the spectra reach the asymptotic regime.

Most notably, the intensity variation of the LUMO 4$a_{1}$ preedge feature in region I of Fig. 1*B* under f-density normalization yields a quantitative measure of donated hydrogen bonds per molecule for the liquid, ice, and gas phases of water. With zero donated bonds for gas and two donated bonds for ice and a linear dependence between the structural parameter average and line intensity, we derive from the f-density normalized prepeak intensities (*SI Appendix*, Table S1) of liquid water an average of 1.74 ± 2.1% donated hydrogen bonds per molecule (for discussion about the error estimate and linear interpolation used, see *SI Appendix*). One should notice that correlation between the area of the preedge peak σ and the number of donated H-bonds used here is based on our work (30), and it resembles the relation of the relative intensity of preedge peak and bond order between the donor H and the acceptor O atoms (figure 1 in ref. 31). The result obtained in this manner is significantly closer to the two donated bonds of ice than the previously derived 1.1 (ref. 11 and *SI Appendix*) bonds per molecule. With the interpolation method used here, for the spectra normalized in area up to 550 eV, the value 1.67 is obtained. We conclude that breaking of H-bonds between ice and liquid water occurs to a lesser degree than concluded in ref. 11.

Quantitative line-intensity–structural-parameter correlation based on a first-principles liquid simulation (30) can now be considered (Fig. 1*D*). For the preedge LUMO 4$a_{1}$ (region I), intensity is anticorrelated to donated (D) and accepted (A) hydrogen bonds, but correlated to the sum angular deviation from tetrahedrality ($\Delta_{a}$) as well as furthest-nearest difference ($\Delta_{d}$) of the closest four neighboring O sites.

For the postedge region III, we observe in f-density normalization a strong rise of spectral intensity going from liquid water toward ice structures and no contributions in gas (Fig. 1*C*). Since the postedge of condensed water resides in the continuum [O1s binding energy in liquid ${BE}_{O1s}$ = 538.21 eV (32)], its interpretation must not foot on bound-state arguments, but can only be attributed to continuum-scattering resonances (shape resonances) reflecting structural order (26). The shape resonances provide direct information about bond length or radius of a coordination shell (33, 34). For a shape resonance at ${\text{hv}}_{in}∼$542 eV, the poles of the electronic wave function in continuum with $E_{kin}$ = ${hv}_{in}-{BE}_{O1s}$ = 542–538.21 eV ∼ 4 eV show characteristic length scales of 3.1 and 4.6 Å, respectively (*SI Appendix*). This makes the shape resonance region sensitive to the first and second solvation shell radii (35) and the corresponding potential barrier height, values of which (from digitization) are presented in *SI Appendix*, Table S2. The interpretation of the postedge (III) as a shape resonance has been proposed to originate from the nearest neighbors (36). We attribute the postedge (III) intensity behavior to be caused by a shape resonance that is due to both first and second solvation shells. This conclusion is supported by matching the solvation shells and their radial-distribution-function peak heights as a measure of the mean barrier height. This continuum scattering resonance in liquid and ices is responsible for the artificial suppression of the preedge when area normalization from 530 to 550 eV is used.

The complete breakdown of the hydrogen-bond network of water in the gas phase is reflected in a rising preedge (I) (Fig. 1 *B* and *C*), whereas the postedge (III) disappears due to loss of solvation-shell order needed for the shape resonance. In the language of quantitative line-intensity–structural-parameter correlation coefficients based on first-principles liquid simulation (30), in Fig. 1*D*, this is expressed as dominant anticorrelation between the shape-resonance intensity in region (III) with the sum angular deviation from tetrahedrality ($\Delta_{a}$) and correlation with donated (D) and accepted (A) hydrogen bonds.

## Resonant Inelastic X-Ray Scattering Spectra

**Fig. 2.**
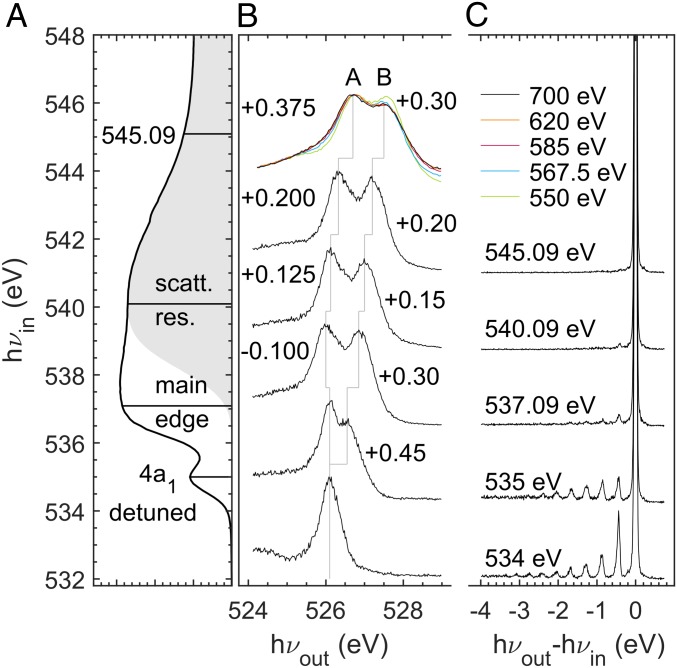
Liquid water at ambient conditions. (*A*) Oxygen 1s X-ray absorption in direct relation to O1s RIXS with sub-natural-linewidth spectral resolution of 50 meV. (*B*) 1$b_{1}$ highest occupied molecular orbital (HOMO) electronic losses at various incident-photon energies normalized to respective maximum value. (*C*) Vibrational losses normalized to main elastic peak height mapping the ground-state potential energy surface along selected coordinates. The shaded area is the contribution of photoionization continuum with an ionization threshold built up from step functions of each of the manifolds of the molecular species in liquid water.

Next, we turn to liquid water by studying O K-edge resonant inelastic X-ray scattering (RIXS) (Fig. 2). We focus on the bound excitations to 4$a_{1}$ LUMO (region I) to states at the main edge and core-ionization continuum through the scattering resonance. In this work, the RIXS spectra were recorded with unprecedented resolving power (>10,000) by using the superadvanced X-ray emission spectrometer (SAXES) (37) at the Advanced Resonant Spectroscopies (ADRESS) beamline (38) of the Swiss Light Source at the Paul Scherrer Institut. Finally, we present X-ray emission spectra (XES) taken at numerous incident energies approaching the sudden core ionization, measured with an instrument of more modest resolution (for complete XES spectra, see *SI Appendix*, Fig. S3).

By comparing the RIXS spectra of electronic loss features (Fig. 2*B*) taken at 545 eV to those excited to the shape resonance at ∼540–542 eV (region III), a noticeable shift of +0.20 eV is observed, due to different coupling and screening of a fast photoelectron and a slow resonantly trapped photoelectron. Trivially, for both continuum excitations, photoionization leads to no vibrational excitations in the quasielastic region (Fig. 2*C*), as the ionized system cannot return to the neutral ground state. We note that XES spectra with differing energy calibrations have been reported (39, 40); we calibrated with respect to data from ref. 39.

As seen in Fig. 2*C*, excitation into the electronic bound-state 4$a_{1}$ LUMO of liquid water yields strong vibrational excitations next to the elastic line. These excitations represent the projection of the core-hole-state-propagated wavepacket back onto the molecular ground-state potential energy surface (41–43). For the main edge, the experimental vibrational progression in liquid water shows significant shortening over the gas phase, a sign of suppression to exhibit the symmetric stretch mode in the liquid environment.

**Fig. 3.**
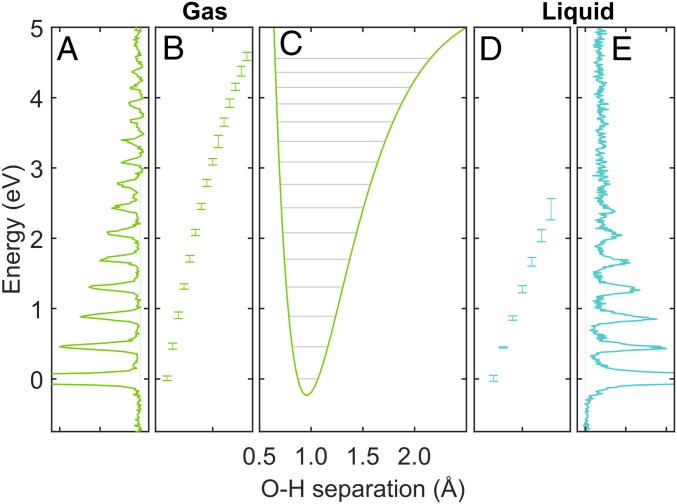
(*A*, *B*, *D*, and *E*) Ground-state vibrational levels along the O–H coordinate of molecular moities present in gas-phase (*A* and *B*) and liquid water at ambient conditions (*D* and *E*) extracted from the vibrational progressions of O1s subnatural linewidth RIXS excited at the 4$a_{1}$ LUMO X-ray absorption resonances, respectively. (*D*) Broadening of vibrational progression in the liquid phase from continuous distribution of molecular configurations. There is no increasing broadening in the spectrum from a single-$H_{2}$O molecule in the gas phase (*B*). The single potential energy surface along the O–H coordinate for the gas phase $H_{2}$O is extracted as a Morse potential (*C*).

In Fig. 3, we show side by side the experimental vibrational losses via the electronic bound-state 4$a_{1}$ LUMO for gas-phase water (Fig. 3*A*) and for liquid water (Fig. 3*D*). The ground-state potential energy surface as a function of O–H distance extracted from experimental RIXS for the gas phase, using a Morse-potential-cut approach as has been used in ref. 43, is shown for gas (Fig. 3*C*). The vibrational progressions for both gas and liquid water show only a single dominant O–H stretch mode. In the gas phase, this mode persists as a distinct peak up to very high vibrational quantum numbers. In the liquid phase, however, broadening toward higher vibrational quantum numbers sets in, which is caused by a statistical distribution of the liquid local environments. No indication for two energetically shifted, distinct O–H stretch frequencies indicative of a two-phase model can be detected.

## X-Ray Emission Spectra

Finally, let us turn to the RIXS electronic losses in Fig. 2*B*, where the 1$b_{1}$ emission line in the water O K-edge XES appears as a double peak in condensed phases (16). This splitting (A and B in Fig. 2*B*) has been promoted as a fingerprint of two distinct structural motifs within the liquid phase (44), which is opposed by arguments of nuclear dynamics causing this effect (18, 19, 45). In the latter view, it is important to realize that the splitting at ionization may have a different origin compared with those of different resonant states due to different core-hole-state potential energy landscapes, and therefore possibly different dynamics.

The XES spectrum taken at 550 eV and above in Fig. 2*B* (for full spectra, see *SI Appendix*, Fig. S3) manifests the photon energy dependence in the continuum, which indicates that the ionized electron still is coupled to the decay. Matching the behavior of the XAS spectra in Fig. 1*C*, at 585 eV and above, convergence of the XES spectral shape is observed, with a resulting spectrum that significantly resembles that recorded for ice recorded by using an X-ray tube (figure 1 in ref. 46) than at lower energies. This finding alone questions the use of the split peak components as indicators of two liquid phases, as this would imply solid ice at liquid-nitrogen temperature to have these phases.

**Fig. 4.**
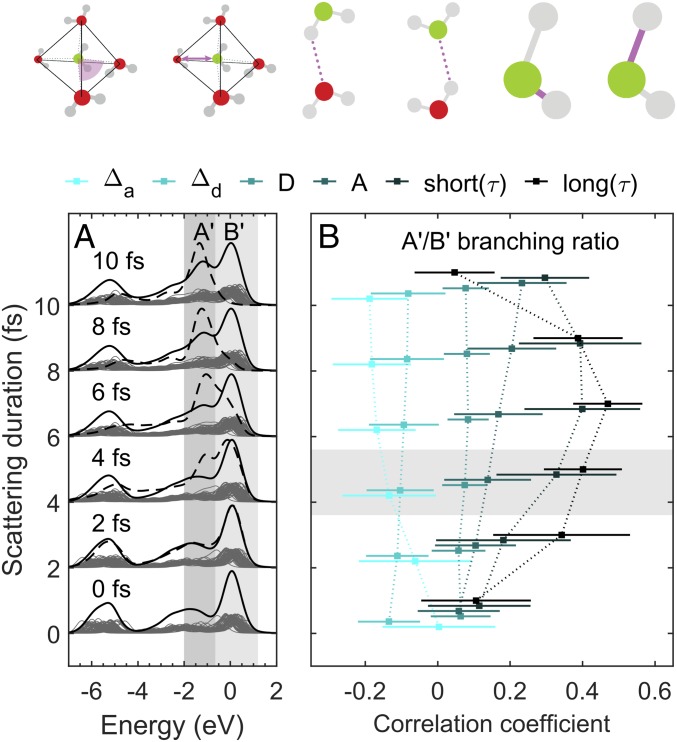
Formation of a split peak in the 1$b_{1}$ HOMO electronic losses from ultrafast molecular relaxation during the femtosecond natural lifetime of the O1s core ionized intermediate state of RIXS in the sudden limit (X-ray emission, XES). (*A*) Molecular dynamics (MD) simulation of O1s RIXS under sudden limit as a function of scattering duration τ= 0, 2, 4, 6, 8, and 10 fs (individual decay-time averaged spectra scaled ×0.2). The instantaneous average is shown as a dashed line. (*B*) Correlation coefficients between the split-peak branching ratio (A′/B′) from core hole dynamics (time-averaged integrated XES spectra) and structural parameters at the site of ionization. The error bars represent 1,000-fold bootstrap resampling. Shown is weak correlation of split-peak intensity sum branching ratio (A′/B′) to angular deviation from tetrahedrality ($\Delta_{a}$), to the furthest-nearest difference ($\Delta_{d}$) for the closest four neighboring O sites, and to donated (D) and accepted (A) hydrogen bonds (the parameters are calculated at the moment of ionization). Long(τ), strongest correlation to the elongation of the long O–H bond during the scattering process; short(τ), stronger correlation to the elongation of the short O–H bond during the scattering process.

Our Bethe–Salpeter equation XES simulations (average 1.88 accepted and donated hydrogen bonds per molecule) account for core-hole dynamics of different durations (Fig. 4*A*). They show that the formation of the lower-energy split component requires core-ionized-state dynamics in the long and the short O–H bonds to take place between ionization and X-ray emission. This dynamical interpretation also explains why slower-moving deuterated samples show a reduced peak A (19, 39, 40, 47–49). For resonant excitations and ionization, the quantitative details of the split peak may be different, as the potential energy surfaces governing the dynamics, in principle, may differ from each other. Still, the dynamic view is consistent with spectra obtained at detuned 4$a_{1}$ resonance, where the electronic loss feature appears as a single line that develops into a double peak when tuned to the 4$a_{1}$ and above. This is understood as an indication of longer effective scattering duration.

The split peak has a weak dependence to underlying structure seen in the branching ratio A/B, similarly to what we have established for the chain-length dependence in liquid alcohols (50). We performed (Fig. 4) a full statistical analysis linking the A′/B′ branching ratio to the continuum RIXS simulation for liquid water. We reveal how A′/B′ increases by increased hydrogen bonding (donated, D; accepted, A) and decreases by increased deviations from tetrahedrality ($\Delta_{a}$, angular; $\Delta_{d}$, distances) of the environment (Fig. 4*B*). These findings are in full agreement with experiments presented here and earlier: The lower-energy component (Fig. 2*A*) of the split peak in water is reduced in higher temperatures of the liquid (19, 47) and increased upon freezing (19, 46). The occurrence of the split peak of water XES itself is a dynamical effect, equivalent to, i.e., alcohols, where the branching ratio picks up some weak but notable statistical trend to structural parameters, as shown in our simulations.

## Conclusions

When putting the information from the three spectroscopies, X-ray absorption, RIXS, and nonresonant X-ray emission, together, we can proceed to conclusions. Analysis of the X-ray absorption across the phase diagram of water using f-density normalization reveals for liquid water 1.74 donated hydrogen bonds per molecule, being closer to the two donated hydrogen bonds in fourfold coordinated tetrahedral ice than previously derived from short-range spectral normalization. In this quantitative normalization, the occurrence of a continuum scattering or shape resonance representing the structural order of the oxygen–oxygen next-neighbor coordination shells in the liquid and ice is established. This shape resonance is absent in gas and supercritical phases, since the number of hydrogen bonds is reduced.

The consequence of this quantitative understanding is that RIXS via the $H_{2}$O LUMO 4$a_{1}$ state is sensitive to all bonding arrangements that might be present in liquid water. Potential-energy-surface mapping with subnatural linewidth RIXS on gas-phase and liquid water finds no indication of two distinct molecular potentials. A split peak in RIXS vibrational progression would be a potential (but not conclusive) indication of two structural motifs. This kind of behavior is not observed at spectral bandwidth of 50 meV. Instead, we observe gradual broadening in a continuous way, which strongly supports the continuum-model description of liquid water.

In nonresonant X-ray emission spectroscopy, the branching of the HOMO 1$b_{1}$ state into a split peak has been promoted as a signature of two structural motives in liquid water. The experimental finding, that in the sudden limit (at high incident energy), the photoelectron decouples from decay, yielding an ice-like emission spectrum, rules out the use of this emission spectrum as evidence for two structural motives in the liquid. This reasoning roots on similarity of the emission spectra and the fact that ice does not have two liquid phases. Additional support is given by a liquid 64-water simulation with periodic boundary conditions, including both structural variation and core-hole-state dynamics on equal footing, being in line with numerous previous simulations. In particular, split-peak branching-ratio relationships show that dynamics play a key role in the formation of the 1$b_{1}$ double peak, with a very weak dependence on the starting structure.

Thus, the findings of X-ray-spectroscopic tools are in full agreement with the continuous-distribution model of liquid water structure equally reported in the vast number of non-X-ray–based investigations of water.

## Materials and Methods

The hard X-ray Raman experiment for liquid water and ice was performed by using the XRS spectroscopy instrument (51) at the beamline ID20 of the European Synchrotron Radiation Facility. The momentum transfer used for detection was q=2.6±0.6 Å^−1^. The scans for the ice sample were performed from below and from above to confirm that radiation damage did not introduce an error in the data (*SI Appendix*, Fig. S2). For the experiment, the liquid water sample was filled into a custom-made flow cell (52), and the ice sample was prepared in situ in a 2-mm quartz capillary continuously cooled by using a cryostream (Oxford cryosystems) at approximately at −88 °C. For both samples, milli-Q water was used. The raw data were handled as described in ref. 53. The intensity integral values in the data are presented in *SI Appendix*, Table S1. There are also problems in using the f-sum rule due to varying completeness of the set of accessible final states (54), here seen as a mismatch between the gas phase and condensed phases. The XAS of Fig. 3 was recorded by using the flat-jet transmission near-edge X-ray absorption fine structure setup (55) at BESSY-II. The ionization step of Fig. 2 was taken from Gaussian-shaped assumed O1s photoline (32) with width from ref. 56.

The RIXS experiment was performed with the SAXES spectrometer (37) at the ADRESS beamline (38) of the Swiss Light Source at Paul Scherrer Institut. The RIXS process proceeds via a core-hole state, thus rendering the technique element-specific and local. Core excitation with subsequent decay into the electronic ground state can furthermore lead to a population of purely vibrational final states. The energy spacing of the obtained vibrational progression allows extraction of the local ground-state potential energy surface (41, 57) The propagation in the core excited-state potential takes the wave packet violently away from the ground-state equilibrium position. Decay of this wavepacket then allows population of ground-state vibrational eigenstates; in particular, is it possible to easily reach the high eigenstates that are not accessible by Raman or IR spectroscopy. This enables reconstruction of the potential energy surface far away from the equilibrium geometry. The dynamics of the core-excited wave packet is state-dependent, which allows for different modes of the system to be probed by selection of the excited state (58).

We used a flow cell separating the sample from the vacuum by a ${Si}_{3}N_{4}$ window of 150-nm thickness with a ∼10-nm Au coating. The energy calibration was based on $O_{2}$ spectrum (41). Due to breakdown of the windows in irradiation, the cell was moved between the spectra. To avoid errors from this procedure, these individual scans were shifted to the same energy by using a fit to the elastic line before joining them. The data in the electronic loss region are presented with larger energy binning for improved statistics.

The XES experiment for photon energies 550 eV and above was performed at the beamline U49-2/PGM-1 in BESSY-II by using the setup described in ref. 59. The XES data were calibrated by using the spectrum at 550.1 eV reported in ref. 39.

## Supplementary Material

Supplementary File
