# Supplementary material for: Compatibility of quantitative X-ray spectroscopy with continuous distribution models of water at ambient conditions
Source: Proc Natl Acad Sci U S A. 2019 Feb 19;116(10):4058–63. doi: 10.1073/pnas.1815701116 (PMC6410789; doi:10.1073/pnas.1815701116)
Supplement: Supplementary File [file pnas.1815701116.sapp.pdf]

PNAS
www.pnas.org

Supplementary Information for

Compatibility of quantitative X-ray spectroscopy with continuous distribution models of water at ambient conditions

Johannes Niskanen, Mattis Fondell, Christoph J. Sahle, Sebastian Eckert, Raphael M. Jay, Keith Gilmore, Annette Pietzsch, Marcus Dantz, Xingye Lu, Daniel E. McNally, Thorsten Schmitt, Vinicius Vaz da Cruz, Victor Kimberg, Faris Gel'mukhanov, and Alexander Föhlisch

A. Föhlisch.
E-mail: alexander.foehlisch@helmholtz-berlin.de

This PDF file includes:

Supplementary text
Figs. S1 to S5
Tables S1 to S3
References for SI reference citations

www.pnas.org/cgi/doi/10.1073/pnas.1815701116

Supporting Information Text

For ambient water (25 C°), an experimental fit performed in Ref. 1 (using short spectra) yields distribution of 15%, 80%, 5% for water molecules with 2,1, and 0 donated hydrogen bonds, respectively. This results in the average of

$$0.15 \times 2 + 0.8 \times 1 + 0.05 \times 0 = 1.1$$

donated (and accepted) H-bonds per water molecule, which differs greatly from the number 1.74 obtained in this work.

Our extraction of the number of donated H-bonds is based on linear interpolation of area of X-ray absorption intensity of pre-edge peak (region I)

$$\sigma = \sigma_{\mathrm{gas}}(1 - aN_d) \qquad [1]$$

with known values of number of donated H-bonds for gas $N_d = 0$ and ice $N_d = 2$. This equation together with areas of the pre-edge peaks for gas, liquid and ice (Table S1) allows to find unknown constant a and results in the value $N_d = 1.74 \pm 2.1\%$ for liquid water under ambient conditions. There is no strict derivation of Eq. 1. However, there is indirect evidence that this linear approximation is a reasonable one.

Indeed, the used linear interpolation is supported by our previous results devoted to the simulation of XAS for 64 water molecules in a $(1.24nm)^3$-cubic cell with periodic boundary conditions (see Ref. 2). Individual XAS spectra were calculated for each oxygen atom of 21 snapshots, sampled with 5-ps intervals by using the Bethe-Salpeter equation and MD simulations. This sampling procedure yields $1344 = 64 \times 21$ spectra for ambient conditions. Fig. S5 depicts the mean area of pre-edge σ for water molecules as a function of the number of donated hydrogen bonds (N_d) from a simulation of liquid water in Ref. 2 together with the individual data points. Only values N_d=0,1,2 are shown.

There are a few definitions of H-bonds (see Ref. (3)). We use the geometrical criteria frequently applied in studies of H-bond networks (see Ref. 1 and Ref. 4). To count N_d we classify a water molecule as an H-bond donor, when the oxygen atoms of the two water molecules are separated by less than 3.5 Å and the $\mathrm{H{-}O_{donor}{-}O_{acceptor}}$ (H-O...O) angle is less than or equal to 30°. The area of pre-edge intensity (σ) was evaluated as the area integral from the XAS spectra in the range [533,535.5] eV. The spectra and the number of donated H-bonds were evaluated for each snapshot and for each molecule separately, which yielded 1344 individual (σ, N_d) pairs, that we here classify with respect to N_d for average pre-edge intensity evaluation. For more details, see Ref. 2.

For different N_d, scatter in the pre-edge intensity is inevitable, because different local structures for selected N_d form a distribution of XAS intensities. The data has 9 points for N_d=0, 208 points for N_d=1, and 1122 points for N_d=2. The remaining 5 points have N_d=3. Fig. S5 displays approximately linear behaviour for the pre-edge mean intensity as a function of N_d, which we use to justify Eq. 1. It is interesting to notice that correlation between σ and N_d presented here resembles the distribution of bond order between the donor H and the acceptor O (see Fig.1 in Ref. 5). In described approach, the natural variation of the transition matrix element in the simulated liquid environment is automatically accounted for (see Ref. 2).

Since we compare spectra from different methods (EELS for gas phase and XRS for condensed phases), we must consider the possibility of the mismatch in our total spectral range integrals (Table S1) to originate from the mismatch in experimental techniques. Here, we derive H-bond number error estimate assuming the mismatch in total integrated intensity to originate from the method.

To arrive in an estimate for error limit, we derive the number of donated H-bonds that would be obtained if the gas-phase spectrum was scaled for its integral to meet the total intensity mean

$$0.5 \times (70.7386 + 70.7064) = 70.7220$$

of the liquid and ice. We note that these two agree rather well. For the gas spectrum to match this intensity, a scaling factor of

$$g = 70.7220/78.5419 \approx 0.90044$$

should be applied to the spectrum, and especially all of its regional integrals. Most notably this yields pre-edge intensity of

$$g \times 4.8801 = 70.7220/78.5419 \times 4.8801 \approx 4.3942$$

to be used in linear interpolation with our assumptions. This interpolation yields the value of 1.7002 and the absolute deviation of $1.7002 - 1.7368 = -0.0366$. Thus the relative error from this consideration reads

$$\frac{|1.7002 - 1.7368|}{1.7368} \approx 0.021 = 2.1\%.$$

However, we are do not have means to assess other possible errors arising from such comparison. Ideally water gas spectra should be recorded in the same experiment as liquid and ice for unbiased comparison. However, it is clear that while X-ray Raman scattering is saturation free, for O K-edge spectra of water gas it is not a feasible experiment with existing synchrotrons and their instrumentation.

Accurate theory of shape resonance is based on multiple scattering theory (6, 7). However, we have densely packed medium in liquid water where one can appeal to concept of coordination shell. In this case the simplest theory which sheds light on the energy position of shape resonances is based on effective potential barrier created by the coordination shell. In this case

we should solve Schrödinger equation for wave function in spherical coordinates. Replacing the wave function $\psi(r)$ by $\chi(r)$: $\psi(r) = \chi(r)/r$ we get the following Schrödinger equation

$$\left(-\frac{1}{2}\frac{d^2}{dr^2} + V(r)\right)\chi(r) = E_{kin}\chi(r), \quad E_{kin} = \frac{k^2}{2}, \qquad [2]$$

where the potential $V(r) = U(r) + l(l+1)/r^2$ for the $l-$th harmonic include the centrifugal potential. We model the confinement of an electron inside of a coordination shell at $r = R$ by the following potential

$$V(r) = \begin{cases} \infty, & r = 0, \\ 0, & 0 < r < R, \\ V_0, & r > R. \end{cases} \qquad [3]$$

In spite of the fact that this model with extensive barrier ignores tunnelling, it allows to get the energy of the shape resonance. Solution of Eq. 3 is straightforward and gives the following equation for the energy of the shape resonance ($E_{kin} < V_0$)

$$\tan(kR) = -\frac{k}{\kappa}, \qquad [4]$$

where $k = \sqrt{2E_{kin}}$ and $\kappa = \sqrt{2(V_0 - E_{kin})}$. This equation comes from the pole of the electronic wave function in the case of the barrier of finite length. The solution of Eq. 4 is as follows

$$kR = n^*\pi, \quad n \le n^* \le n + \frac{1}{2}. \qquad [5]$$

Here n is integer. Apparently $n^* = n$ when $V_0 = \infty$ while $n^* = n + 1/2$ when the energy E_{kin} approaches V_0. Now we are in stage to write equation for the radius of coordination shell

$$R \approx n^* \times \sqrt{\frac{37.56}{E(eV)}}\text{Å}. \qquad [6]$$

In the case of our interest $E_{kin} \approx 4$ eV. Thus

$$R \approx 3.06 \times n^*\text{Å}, \quad E_{kin} = 4\ eV. \qquad [7]$$

Therefore, we estimate the radius R of the coordination spheres for the shape resonance near $E_{kin} = 4$ eV

$$R \approx 1.53\text{Å}, \quad 3.06\text{Å}, \quad 4.59\text{Å}, \quad 6.12\text{Å} \qquad [8]$$

for effective quantum numbers $n^* = 0.5$, 1, 1.5, and 2, respectively.

Eq. 5 shows the general property of the peak position of shape resonance

$$E_{kin} = \frac{\mathrm{C}}{R^2}. \qquad [9]$$

This correlation between the shape resonance and position of the effective barrier is universal and is widely used to extract the bond length from the experimental data (8, 9). One should notice that the constant C is sensitive to the system (diatomic molecule, polyatomic molecules, liquids etc).

The gas and liquid lines of the phase diagram in Figure 1 are from Refs. 10, 11. For the phase diagram, the gray coexistence curves between the triple points of ices (11–17) were obtained by Clausius equation as a boundary value problem with fixed $\Delta S/\Delta V$.

The experimental RIXS spectrum contained sidepeaks due to X-ray optics that were subtracted as follows: (i) a constant background (the mean between -5.0 and -3.0 eV in loss scale) was subtracted; (ii) the spectra were normalized to highest quasielastic to be 1.0 in height; (iii) a pseudo-Voigt lineshape for elastic and the sidepeak were fitted in the range between -5.0 and 0.2 eV in loss, points up to 2.5 in intensity are considered; (iv) the side peak was mirrored to the positive-loss side; (v) subtraction of these fitted side peak shapes was performed. The fitting parameters do not have a simple physical correspondence, and the procedure is justified by elimination of the side-peak on the anti-Stokes side of the spectrum, which is present due to spectrometer optics.

Line positions and widths from the RIXS spectra were defined by a fit using Voigt profiles with a shared Lorentzian contribution, which was varied in the process. The Gaussian broadenings σ of the lines were treated as independent parameters, and are reported as widths in Figure 3. The elastic line was included to the fit only up to the level of other lines, meaning that the top part of the true elastic line was ignored. This is motivated by the line shape not being a Voigt profile.

For the obtained line positions, we performed a fit using the effective mass of μ=0.94 a.m.u. in a one-dimensional Morse potential

$$V(r) = D_e(1 - \mathrm{e}^{-a(r-r_0)})^2 \qquad [10]$$

with eigenvalues

$$E_i = h\nu(i + 1/2) - \frac{(h\nu(i+1/2))^2}{(4D_e)}, \quad \nu = \frac{a\sqrt{2D_e/m}}{2\pi} \qquad [11]$$

to obtain parameters D_e and a. The position of the minimum r_0 is not obtainable from the data and was given the value 0.95 Å, when drawing the potential energy curve for gas.

The XES simulations were carried out in three steps. First, we obtained a snapshot from *ab initio* molecular dynamics (AIMD) of 64 water molecules (structural simulation). Second, starting from this snapshot we carried out 64 AIMD runs simulating the effect of a core hole at each particular oxygen site in turn (dynamics simulation), with a specific pseudopotential designed for the task (18). These 64 AIMD runs simulate the violent atomistic dynamics after the core-level ionization of a particular oxygen atom. Last, emission spectra were evaluated along each of these 64 dynamics simulation trajectories (spectrum simulation). All AIMD simulations employed the gradient-corrected BLYP (19, 20) density functional and a 85 Ry kinetic energy cut-off for the plane wave expansion of the Kohn-Sham wave-functions in combination with a pseudopotential description. The structural simulation utilised Car-Parrinello molecular dynamics (21) (CPMD) for simulation a cubic cell (cubic cell with a=12.4170 Å) of 64 molecules within the CPMD software (version 3.11) (22). The system was equilibrated for at least 6 ps (starting from classical force field models) at 300 K in the NVT ensemble with a time-step of 0.1 fs and a fictitious electron mass of 500 au. For hydrogen a local pseudo-potential parametrized with one Gaussian was used. For oxygen, the norm-conserving pseudo-potentials were of Troullier-Martins type (23), expressed in the Kleinman-Bylander form (24). The dynamics simulation was carried by continuing the ground state CPMD using the Born-Oppenheimer AIMD.

The XES spectrum simulation was done at selected times on the 64 core-hole-dynamics trajectories using the Bethe-Salpeter equation (BSE) formalism as implemented in the OCEAN code (25, 26). For the XES calculations the electronic ground state calculations were performed using the Quantum ESPRESSO program package (27) and norm-conserving pseudopotentials obtained from the ABINIT distribution (28). For the calculation of the ground state electron density we used Γ-point sampling, while a $2 \times 2 \times 2$ k-point mesh and 1600 bands were used for evaluation of the final scattering states. Wave functions were truncated beyond a cutoff of 70 a.u. The sampling along the core-hole induced dynamics trajectory was performed at 2 fs intervals up to 10 fs, and a broadening with a gaussian of 0.5 eV full-width-at-half-maximum was applied to the spectrum. Because the decay-rate (and the relative contribution of XES spectra of later times) follows the same exponential law as the core-hole-state, we computed the XES $\sigma(\tau)$, accumulated up to time τ as follows

$$\sigma(\tau) = \int_0^\tau \sigma_{\text{ins}}(t) e^{-\frac{t}{\tau_{\text{core}}}} dt; \quad \tau_{\text{core}} = \frac{1}{FWHM} = \frac{0.658}{0.16eV} = 4.11\text{fs} \qquad [12]$$

The energy scale is relative to the ground state of the ion, and we averaged over all oxygen sites of the simulation box. The results with different upper limit τ for the integral are depicted in Figure 4, where the instantaneous averaged emission spectra are also shown.

In the analysis of XES, we refer to the structural parameters at the moment of ionization (before the core-ionized dynamics), except for bond lengths that are evaluated at the indicated time. The error limit is based on the standard deviation in 1000-fold bootstrap re-sampling technique.

Supporting data

The short XRS spectra were normalized to the mean value in the range 548–550 eV, and are depicted with f-density normalization and with f-sum rule normalization in Fig. S1. The long spectra were normalized to the mean value in the range 580–585 eV, and are depicted in Fig. S2 and the corresponding intensity integral values are presented in Table S1. The XES spectra recorded photon energies 550 eV and above are shown in full in Figure S3. Tabulated values for the peak positions and heights of the radial distribution functions of water in some condensed phases from Ref. 29 are given in Table S2. The average structural parameters from the XES simulation are given in Table S3. The complete correlation coefficient data of Ref. 2 is presented in Figure S4. Figure S5 shows the relation of the mean pre-peak intensity to N_d from simulations (2).

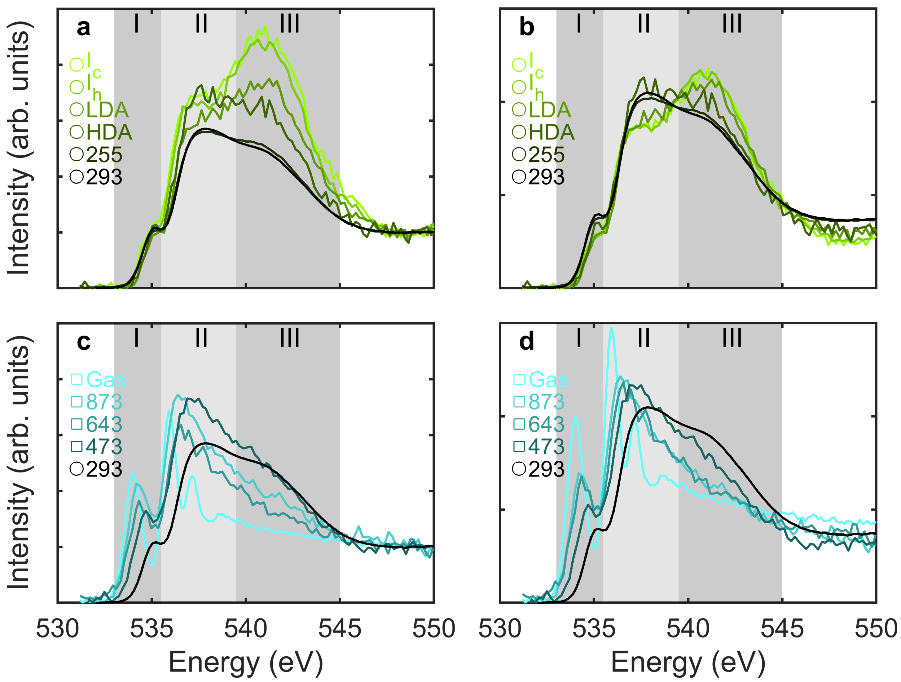

Fig. S1. Previously reported data (30–33) with different normalizations. (a) condensed phases with ionization cross section at 550 eV, (b) condensed phases with area between 531.2 eV and 550 eV, (c) gas phase and supercritical with ionization cross section at 550 eV, and (d) gas phase and supercritical with area between 531.2 eV and 550 eV.

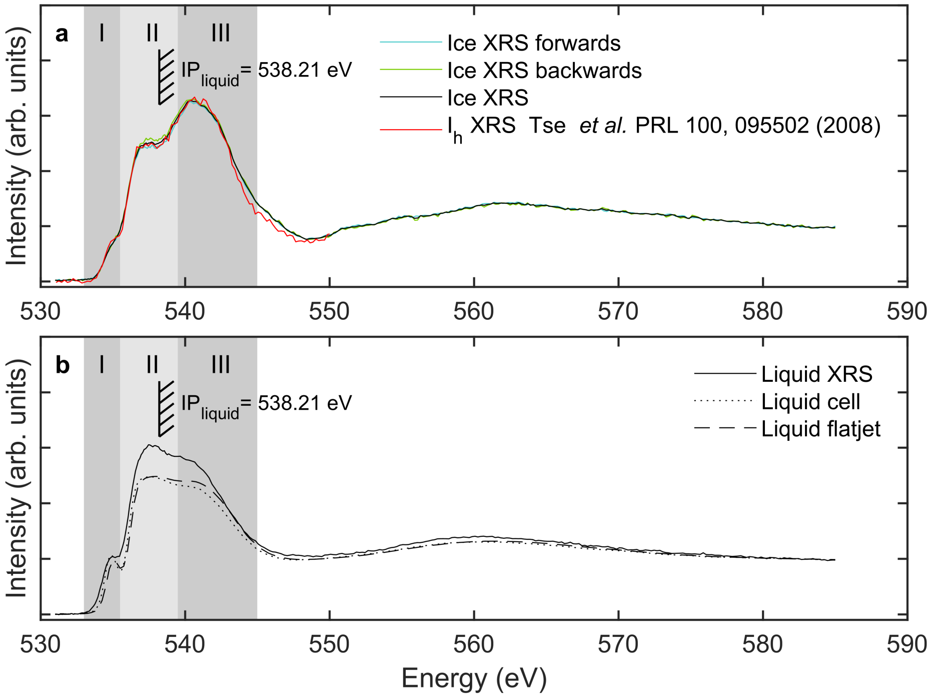

Fig. S2. The scans from below (upwards in energy) and above (downwards in energy) for the ice sample to study the effect of radiation damage on the sample (a). The spectra are consistent with each other and with ice I_h reference, which means that significant radiation damage does not take place. Core-excitation spectra of liquid water recorded with different methods (b).

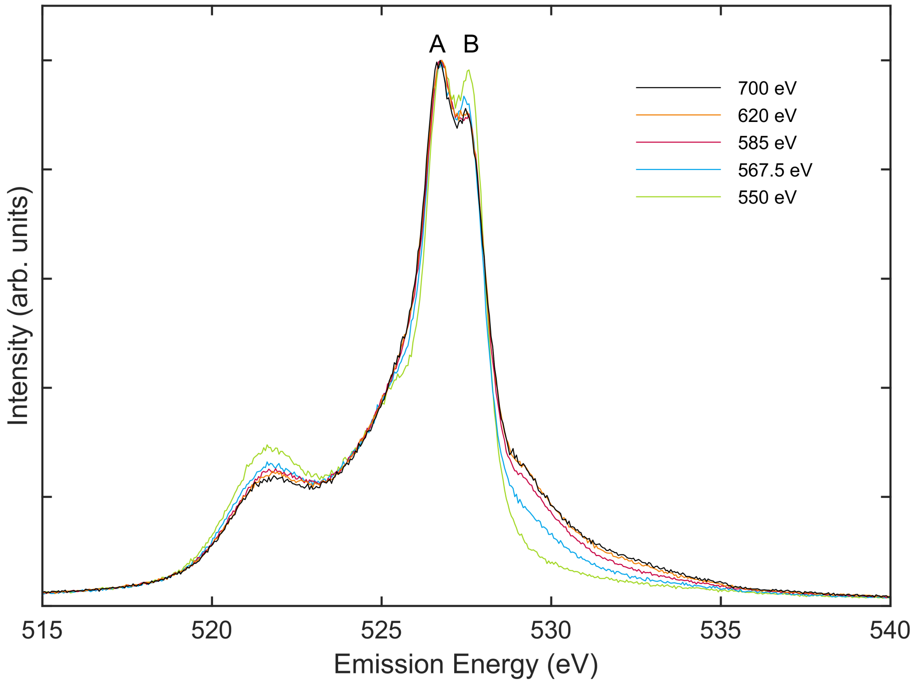

Fig. S3. XES as a function of incident photon energy, as recorded from a liquid jet. The effect of the photoelectron coupled to the decay converges.

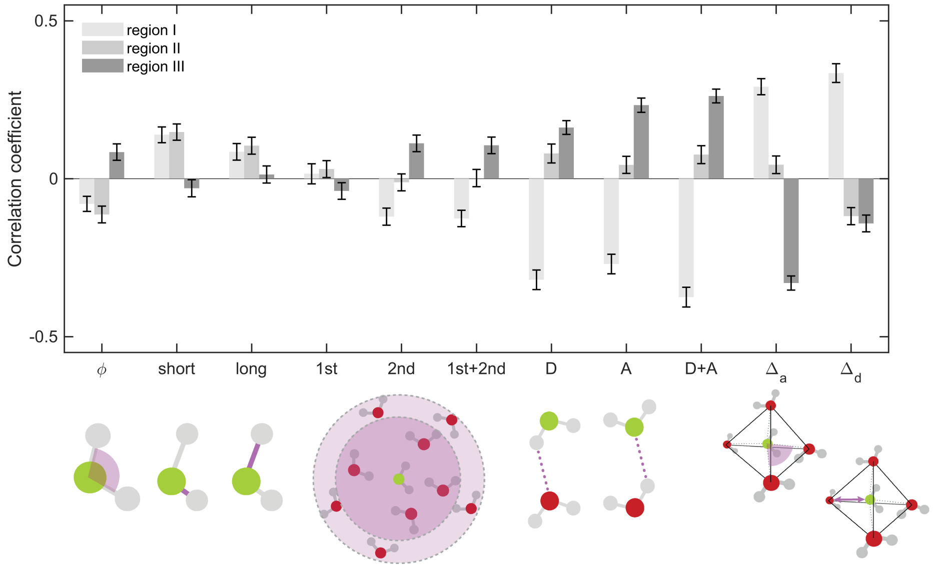

Fig. S4. Line-intensity–structural-parameter correlation coefficients based on first principles liquid simulation for the spectral regions I-III in the O K-edge excitation spectrum (from Ref. (2)): Molecular bond angle (ϕ), bond lengths (short, long), solvation shell occupation (1st, 2nd, 1s+2nd), donated (D) and accepted (A) hydrogen bonds and their sum (D+A), sum angular deviation from tetrahedrality (Δ_a) and furthest-nearest difference (Δ_d) for the closest four neighboring O sites. The hydrogen-bonding and deviation-from-tetrahedrality parameters show stronger correlation with the intensities than internal structural parameters or solvation-shell occupation numbers.

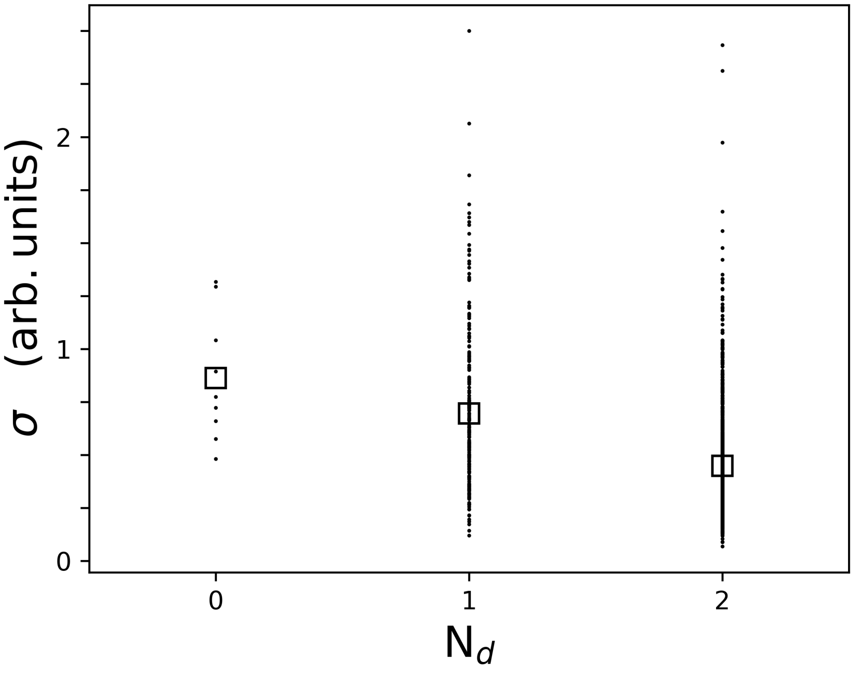

Fig. S5. The area σ of the pre-edge peak (region I) as a function of the number of donated hydrogen bonds (N_d) from Ref 2. The individual points of the line-intensity–N_d data show scatter due to statistical distribution. Black square markers indicate the mean pre-edge peak intensity with each value of N_d, and the marker dots indicate the individual data points in the simulation.

Table S1. The intensity integral values in the long spectra of water in gas, liquid and ice when using the f-density normalization with asymptotic value of 1/eV for df/dE.

	I	II	III	total
gas	4.8801	11.9065	10.4986	78.5419
liquid	1.4256	10.4493	11.8660	70.7386
ice	0.9021	9.2111	14.3835	70.7064

Table S2. First and second solvation shell radii in liquid water and ices in Å from Ref 29, that are directly characteristic to the length scale of the second and third nodes of the scattered electron wave function in the shape resonance (3.1 Å, 4.6 Å). The corresponding peak heights of the radial distribution function are given in parenthesis.

	Liq.	HDA	LDA	$\mathrm{I_h}$
1st	2.8 (2.9)	2.7 (3.0)	2.7 (4.7)	2.8 (3.8)
2nd	4.3 (1.1)	3.9–4.3 (1.2)	4.5 (1.8)	4.5 (2.5)

Table S3. The average values of structural parameters in the XES simulation.

D	A	Δ_a	Δ_d
1.8750	1.8750	106.1293	0.4484

References

1. Wernet P, et al. (2004) The structure of the first coordination shell in liquid water. *Science* 304:995–999.
2. Niskanen J, et al. (2017) Disentangling structural information from core-level excitation spectra. *Physical Review E* 96:013319.
3. Santra B, Jr. RAD, Martelli F, Car R (2015) Local structure analysis in ab initio liquid water. *Molecular Physics* 113(17-18):2829–2841.
4. Kumar R, Schmidt JR, Skinner JL (2007) Hydrogen bonding definitions and dynamics in liquid water. *The Journal of Chemical Physics* 126(20):204107.
5. Fernández-Serra MV, Artacho E (2006) Electrons and hydrogen-bond connectivity in liquid water. *Phys. Rev. Lett.* 96(1):016404.
6. Mazalov LN, Gel'mukhanov FK, Chermoshentsev VM (1974) Effects of multiple scattering in the x-ray absorption spectra of molecules and solids. *Journal of Structural Chemistry* 15:975–982.
7. Dehmer JL, Dill D, Wallace S (1979) Shape-resonance-enhanced nuclear-motion effects in molecular photoionization. *Phys. Rev. Lett.* 43(14):1005–1008.
8. Tse J, Tan K, Chen J (1990) Oxygen k-edge xanes of crystalline and amorphous ice. *Chemical Physics Letters* 174(6):603 – 608.
9. Stöhr J (1992) *NEXAFS spectroscopy.* (Springer-Verlag, Berlin).
10. Wagner W, PrußA (2002) The IAPWS formulation 1995 for the thermodynamic properties of ordinary water substance for general and scientific use. *Journal of Physical and Chemical Reference Data* 31(2):387–535.
11. The International Association for the Properties of Water and Steam (2011) Revised release on the pressure along the melting and sublimation curves of ordinary water substance IAPWS R14-08. http://www.iapws.org/relguide/MeltSub2011.pdf (accessed 4. 8. 2017).
12. Bridgman PW (1912) Water, in the liquid and five solid forms, under pressure. *Proceedings of the American Academy of Arts and Sciences* 47:441–558.
13. Yen F, Chi Z (2015) Proton ordering dynamics of h2o ice. *Phys. Chem. Chem. Phys.* 17(19):12458–12461.
14. Mercury L, Vieillard P, Tardy Y (2001) Thermodynamics of ice polymorphs and 'ice-like' water in hydrates and hydroxides. *Applied Geochemistry* 16(2):161–181.
15. Salzmann CG, Radaelli PG, Mayer E, Finney JL (2009) Ice XV: A new thermodynamically stable phase of ice. *Phys. Rev. Lett.* 103(10):105701.
16. Song M, Yamawaki H, Fujihisa H, Sakashita M, Aoki K (2003) Infrared investigation on ice VIII and the phase diagram of dense ices. *Phys. Rev. B* 68(1):014106.
17. Goncharov AF, et al. (2005) Dynamic ionization of water under extreme conditions. *Phys. Rev. Lett.* 94(12):125508.
18. Cavalleri M, Odelius M, Nordlund D, Nilsson A, Pettersson LGM (2005) Half or full core hole in density functional theory x-ray absorption spectrum calculations of water? *Phys. Chem. Chem. Phys.* 7:2854–2858.
19. Becke AD (1988) Density-functional exchange-energy approxiation with correct asymptotic behavior. *Physical Review A* 38:3098–3100.
20. Lee C, Yang W, Parr RG (1988) Development of the colle-salvetti correlation-energy formula into a functional of the electron density. *Physical Review B* 37:785–789.
21. Car R, Parrinello M (1985) Unified approach for molecular dynamics and density-functional theory. *Physical Review Letters* 55:2471–2474.
22. CPMD (1990–2019). http://www.cpmd.org/, Copyright IBM Corp 1990–2019, Copyright MPI für Festkörperforschung Stuttgart 1997–2001.
23. Troullier N, Martins JL (1991) Efficient pseudopotentials for plane-wave calculations. *Physical Review B* 43:1993–2006.
24. Kleinman L, Bylander DM (1982) Efficacious form for model pseudopotentials. *Physical Reviev Letters* 48:1425–1428.
25. Vinson J, Rehr JJ, Kas JJ, Shirley EL (2011) Bethe-salpeter equation calculations of core excitation spectra. *Phys. Rev. B* 83(11):115106.
26. Gilmore K, et al. (2015) Efficient implementation of core-excitation bethe–salpeter equation calculations. *Computer Physics Communications* 197:109 – 117.
27. Giannozzi P, et al. (2009) Quantum espresso: a modular and open-source software project for quantum simulations of materials. *Journal of physics: Condensed matter* 21(39):395502.
28. Gonze X, et al. (2009) Abinit: First-principles approach to material and nanosystem properties. *Computer Physics Communications* 180(12):2582–2615.
29. Finney JL, Hallbrucker A, Kohl I, Soper AK, Bowron DT (2002) Structures of high and low density amorphous ice by neutron diffraction. *Phys. Rev. Lett.* 88(22):225503.
30. Tse JS, et al. (2008) X-ray raman spectroscopic study of water in the condensed phases. *Phys. Rev. Lett.* 100(9):095502.
31. Lehmkühler F, et al. (2016) Intramolecular structure and energetics in supercooled water down to 255 K. *Phys. Chem. Chem. Phys.* 18(9):6925–6930.
32. Sahle CJ, et al. (2013) Microscopic structure of water at elevated pressures and temperatures. *Proc. Nat. Acad. Sci.* 110:6301–6306.
33. Ishii I, McLaren R, Hitchcock AP, Robin MB (1987) Inner-shell excitations in weak-bond molecules. *The Journal of Chemical Physics* 87:4344.
